# From skills to smart care: The mediating role of digital literacy in the relationship between digital competence and medical artificial intelligence readiness among nursing students

**DOI:** 10.1371/journal.pone.0359992

**Published:** 2026-10-15

**Authors:** Shaimaa Mohamed Amin, Heba Emad El‑Gazar, Sameer A. Alkubati, Mohamed Ali Zoromba, Heba Sobhy Mohamed, Mohammed Adel Abd Elhafeez Elbakry, Ahmed Farghaly Tawfik, Mohamed Hussein Ramadan Atta, Abeer Abd Elaziz Madian, Haitham Mokhtar Mohamed Abdallah, Hanadi Dakhilallah, Azza Abd Elrazek Baraka

**Affiliations:** 1 Department of Community, Psychiatric, and Mental Health Nursing, College of Nursing, Qassim University, Buraydah, Saudi Arabia; 2 Community Health Nursing Department, Faculty of Nursing, Damanhour University, Damanhour, Egypt; 3 Nursing Services Department, Qassim University Medical City, Buraydah, Saudi Arabia; 4 Department of Nursing Administration and Education, College of Nursing, Imam Mohammad Ibn Saud Islamic University (IMSIU), Riyadh, Saudi Arabia; 5 Department of Medical Surgical Nursing, College of Nursing, University of Ha’il, Ha’il City, Saudi Arabia; 6 College of Nursing, Prince Sattam bin Abdulaziz University, Al-Kharj, Saudi Arabia; 7 Psychiatric and Mental Health Nursing Department, Faculty of Nursing, Mansoura University, Mansoura, Egypt; 8 Nursing Administration Department, Faculty of Nursing, Zagazig University, Zagazig, Egypt; 9 Public Health Nursing Department, College of Nursing, Northern Border University, Arar, Saudi Arabia; 10 Nursing Administration, Faculty of Nursing, Beni-Suef University, Beni-Suef, Egypt; 11 Nursing Department, College of Applied Medical Sciences, Prince Sattam Bin Abdulaziz University, Wadi Addawasir, Saudi Arabia; 12 Psychiatric and Mental- Health Nursing Department, Faculty of Nursing, Alexandria University, Alexandria, Egypt; 13 College of Nursing, Medical Surgical Department, Jouf University, Sakaka, Saudi Arabia; 14 College of Nursing, King Saud Bin Abdulaziz University for Health Sciences, Al-Ahsa, Saudi Arabia; 15 King Abdullah International Medical Research Center, Al-Ahsa, Saudi Arabia; 16 Critical Care and Emergency Nursing Department, Faculty of Nursing, Alexandria University, Alexandria, Egypt; University of Setif 1, ALGERIA

## Abstract

The rapid integration of artificial intelligence (AI) in healthcare requires nurses to possess strong digital competence, advanced digital literacy, and readiness to engage with AI technologies. Despite this need, the ways in which digital competence influences AI readiness, and the mediating role of digital literacy, remain underexplored. This descriptive cross-sectional study examined 500 undergraduate nursing students from two Universities for Health Sciences in Saudi Arabia and Egypt using validated measures of digital competence, digital literacy, and medical AI readiness. Results indicated strong positive correlations between digital competence, digital literacy, and AI readiness, with digital literacy partially mediating the effect of digital competence on AI readiness. These findings highlight the importance of integrating ethical, reflective, and AI-focused digital literacy alongside technical skills in nursing education, ensuring that future nurses are prepared to use medical AI effectively and safely, thereby supporting clinical decision-making and improving quality of care.

## 1. Introduction

The rapid advancement of digital technologies has fundamentally transformed healthcare systems, introducing data-driven decision-making, machine learning–assisted diagnostics, and smart clinical interventions. Among these developments, artificial intelligence (AI) has emerged as a transformative tool with significant potential to enhance clinical care, operational efficiency, and health informatics [1]. However, effective AI integration requires not only technological infrastructure but also a workforce that is cognitively, technically, and ethically prepared. As the largest segment of the healthcare workforce, nurses must be ready to engage meaningfully with AI applications, highlighting the importance of evaluating AI readiness among nursing students [2].

The successful adopxtion of AI is strongly influenced by digital competence, a multidimensional construct encompassing technical skills, problem-solving, information management, and digital communication abilities [3]. In nursing education, digital competence supports academic achievement and facilitates students’ transition into technologically advanced clinical environments, forming the foundation for more advanced competencies such as AI utilization [4]. Nevertheless, digital competence alone may not fully prepare students for the complexities of AI-driven healthcare, prompting investigation into intermediary factors that may strengthen this relationship [5].

Digital literacy has been proposed as a key mediating construct linking digital competence to AI readiness. Digital literacy extends beyond technical proficiency to include critical thinking, ethical awareness, information credibility, cyber hygiene, and responsible technology use [6,7]. In AI-integrated healthcare contexts, nurses must not only operate digital systems but also interpret AI outputs, recognize their limitations, and communicate insights effectively. Therefore, digital literacy may enhance the translation of digital competence into practical readiness for AI implementation [3,8].

Empirical studies support the role of digital competence in shaping positive attitudes toward AI and digital health transformation among nursing students [9]. Attitudinal readiness and experiential exposure to digital technologies also play a critical role in AI readiness, as positive attitudes increase acceptance and willingness to engage with AI tools, while hands-on experience with digital systems enhances confidence and reduces perceived barriers to AI adoption in clinical practice [10].

Recent evidence further highlights that nursing students’ readiness, literacy, and attitudes toward medical artificial intelligence are closely interrelated, emphasizing the importance of AI literacy in strengthening preparedness for clinical AI applications [11]. In addition, qualitative findings from nursing faculty experiences indicate that the integration of tools such as ChatGPT is reshaping teaching and learning processes in nursing education, underscoring the growing need for structured AI integration within curricula [12]. High levels of digital competence have also been linked to academic motivation and lifelong learning, both essential components of AI readiness [3]. However, the relationship between digital competence and AI readiness is not always direct and is influenced by contextual and demographic factors such as prior digital exposure, frequency of technology use, and digital literacy levels. Furthermore, experiential engagement with digital tools, including repeated use of digital platforms and exposure to technology-enhanced learning environments, strengthens students’ preparedness for AI integration in healthcare practice [13].

Fostering digital literacy in nursing education extends beyond teaching technical skills and includes developing awareness of AI capabilities and limitations, ethical considerations such as data privacy and algorithmic bias, and critical evaluation of AI-generated information [14]. Despite its importance, many nursing curricula continue to emphasize traditional clinical skills, resulting in a gap between educational preparation and the technological demands of contemporary healthcare [15].

Constructivist learning theories emphasize that digital competence, digital literacy, and AI readiness develop most effectively through active engagement and real-world learning experiences [16]. Accordingly, integrating AI-related content within broader digital education frameworks is essential for preparing future-ready nurses [17,18]. Ethical considerations further reinforce the role of digital literacy, as AI use in healthcare raises concerns related to autonomy, accountability, and data governance [19]. Enhancing digital literacy therefore supports not only technical preparedness but also ethical competence in AI-augmented clinical practice [20,21].

To address gaps in the existing literature, this study examines the mediating role of digital literacy in the relationship between digital competence and medical AI readiness among undergraduate nursing students. Using a descriptive cross-sectional design and validated instruments including the Digital Competence Scale, Digital Literacy Scale, and the Medical Artificial Intelligence Readiness Scale for Medical Students the study applies structural equation modeling to test the proposed relationships [22–24]. The findings aim to extend theoretical models of digital preparedness and provide practical guidance for integrating ethical, reflective, and competency-based digital education into nursing curricula.

## 2. Materials and methods

### 2.1. Aim

This study aims to examine the mediating role of digital literacy in the relationship between digital competence and medical AI readiness among undergraduate nursing students.

### 2.2. Design

This study utilized a descriptive cross-sectional design and was conducted in accordance with the STROBE (Strengthening the Reporting of Observational Studies in Epidemiology) guidelines [25].

### 2.3. Settings and sample

The study was conducted at two universities for health sciences in Saudi Arabia and Egypt. Both institutions are well-established academic institutions that provide undergraduate nursing education aimed at preparing students for modern clinical practice. Each college includes several academic departments covering a range of nursing specialties.

Participants were selected based on predefined inclusion and exclusion criteria to ensure the relevance, accuracy, and credibility of the study results. Inclusion criteria consisted of undergraduate nursing students enrolled in any academic year at the Colleges of Nursing who had completed at least one semester and were willing to participate voluntarily. Students were required to have basic digital access (e.g., access to a smartphone, tablet, or computer) and the ability to comprehend and complete the study questionnaire in Arabic or English. Exclusion criteria included students who were on academic leave, those enrolled in bridging or external nursing programs, and individuals who had previously participated in specialized training programs focused exclusively on artificial intelligence to reduce exposure bias and ensure a more homogeneous baseline AI knowledge, allowing clearer assessment of the study relationships.

To determine the appropriate sample size for this study, we employed G*Power software (ersion 3.1.9.7), developed by Faul et al. (2007) [26]. The calculation was based on an anticipated effect size of 0.15, a significance level (alpha) of 0.05, and a desired statistical power of 0.90. Using these parameters, along with data from prior research, the software indicated that a minimum of 459 participants was necessary. To account for potential non-responses, the sample size was increased to 510. However, ten students declined to participate, resulting in a final sample of 500 students.

The flowchart illustrates the participant recruitment and selection process, including the proportional selection of students from each study site to ensure representation across all academic years.There were about 10,200 eligible nursing students in the combined sampling period from both colleges. Every 20th student on a predetermined list of eligible students was chosen using a systematic random sample process at each university with a sampling interval of 20. This method produced a preliminary sample of 510 students. 500 nursing students made up the final sample after 10 students who rejected to participate were removed. The participant recruitment and selection process is shown in Fig 1.

**Fig 1:**
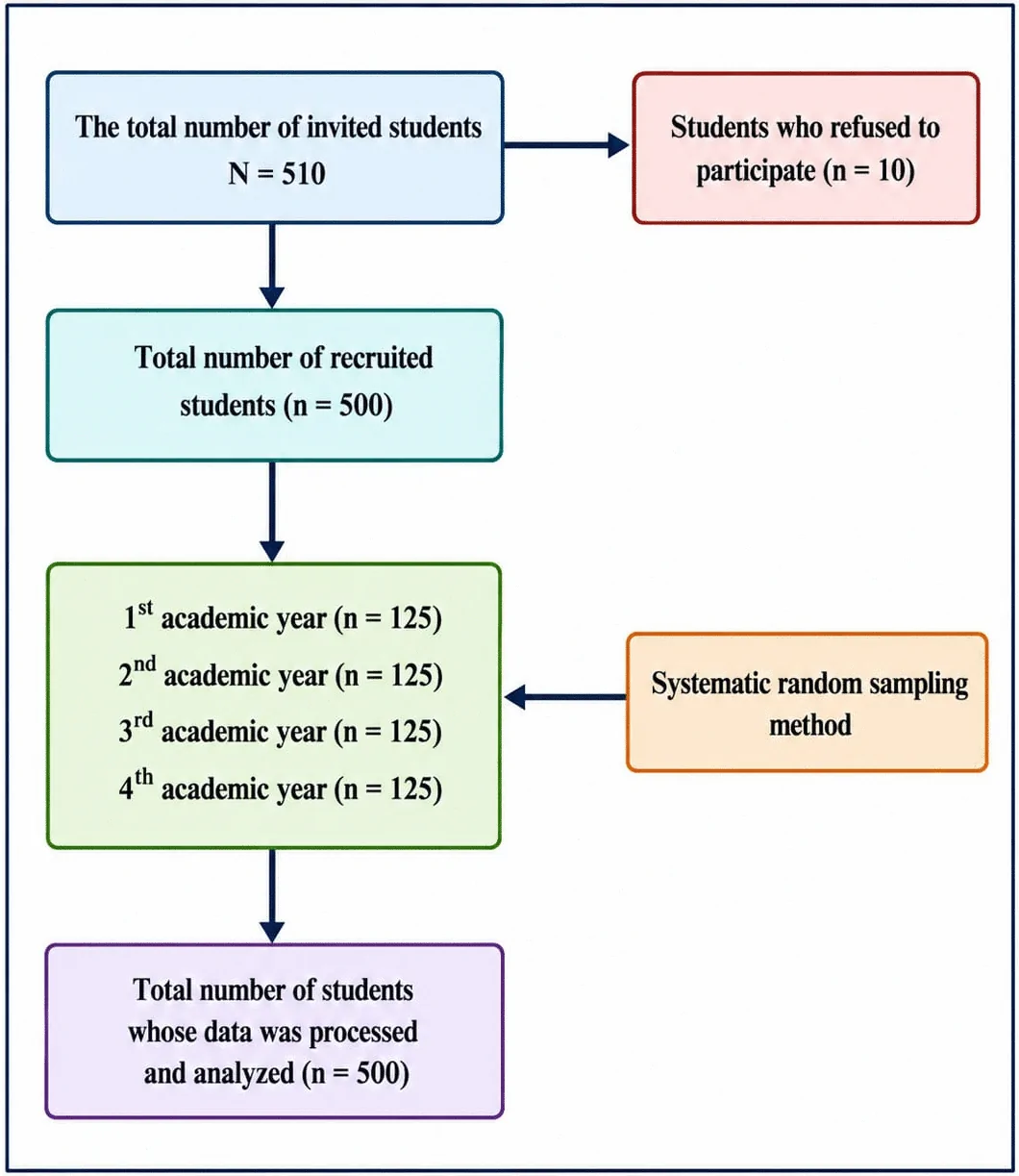
Participant recruitment and selection.

Participant recruitment and selection across the two study sites resulted in a final sample of 500 nursing students.

### 2.4. Data collection tools and methods

A structured demographic questionnaire was designed to obtain baseline information about the participants. The form collected data on age, gender, occupation, residence, family structure, and monthly family income. It also included parents’ educational attainment, students’ current grade point average (GPA), previous experience with artificial intelligence applications in healthcare, frequency of digital device use, home internet availability, and level of interest in innovative healthcare technologies. These variables were gathered to create a detailed participant profile and to explore possible associations with digital competence, digital literacy, and AI readiness.

The Students’ Digital Competence Scale, developed by Tzafilkou and colleagues (2022) [27], measures digital capabilities among university students. The instrument consists of 28 items grouped into six domains: searching and accessing information; developing and modifying content; communication and collaboration; storage and management; evaluation; and protection. Responses were assessed using a five-point Likert scale ranging from 1 (strongly disagree) to 5 (strongly agree), where higher scores reflect greater levels of digital competence. The original scale demonstrated satisfactory internal consistency, as indicated by factor loadings above 0.50 and acceptable Cronbach’s alpha coefficients. Construct validity was examined through confirmatory factor analysis (CFA), which indicated an acceptable model fit. Model adequacy was evaluated using several goodness-of-fit indices, including chi-square statistics, normed fit index (NFI), and root mean square error of approximation (RMSEA).

Reliability testing in this study produced a Cronbach’s alpha of 0.91, which shows good internal consistency. The Digital Literacy Scale (DLS), developed by Avinç and Doğan (2024) [22], evaluates nursing students’ competencies in accessing, analyzing, producing, and communicating information through digital technologies. The tool is particularly relevant for technology-enhanced and blended learning environments. The instrument consists of 20 items evaluated on a four-point Likert scale ranging from 1 (strongly disagree) to 4 (strongly agree). Total scores range between 20 and 80, with higher scores reflecting greater digital literacy abilities.

The original scale demonstrated excellent psychometric properties. Internal consistency was high, with Cronbach’s alpha and omega coefficients indicating strong reliability. Factor analysis revealed substantial loadings across items, explaining over 70% of the variance. Measures of sampling adequacy, including the KMO statistic and Bartlett’s test, confirmed the suitability of the data for factor analysis.

The Medical Artificial Intelligence Readiness Scale for Medical Students (MAIRS-MS), developed by Karaca and colleagues (2021) [23], measures students’ preparedness to integrate artificial intelligence technologies into clinical practice. The instrument was designed to evaluate readiness in response to the expanding role of AI in healthcare. The MAIRS-MS was selected due to its strong theoretical foundation in assessing AI readiness (cognition, ability, vision, and ethics), and because no fully validated AI readiness scale specifically designed for nursing students was available at the time of the study. The scale has been widely used in health education research and was considered suitable for adaptation in the nursing context. It comprises 22 items distributed across four domains: cognition (knowledge of AI concepts), ability (practical skills in using AI tools), vision (attitudes toward future AI integration), and ethics (awareness of ethical considerations). Items are rated on a five-point Likert scale (1 = strongly disagree to 5 = strongly agree), with higher scores indicating greater readiness.

Psychometric evaluation of the original instrument demonstrated a clear four-factor structure explaining a substantial proportion of total variance. Indicators such as KMO values and significant Bartlett’s tests confirmed sampling adequacy. Confirmatory factor analysis supported acceptable model fit based on indices including RMSEA, CFI, and GFI. The overall Cronbach’s alpha indicated strong internal consistency, with acceptable reliability across subscales. In the present study, reliability analysis produced a Cronbach’s alpha of 0.91.

All instruments (MAIRS-MS, DLS, and the Students’ Digital Competence Scale) were translated into Arabic by bilingual specialists proficient in both English and Arabic. Emphasis was placed on linguistic precision and cultural adaptation. A back-translation process was subsequently conducted to verify equivalence with the original versions and resolve discrepancies. Face validity was evaluated by expert reviewers who examined clarity, cultural relevance, and conceptual alignment. Feedback from a small group of students was also obtained to ensure item comprehensibility and appropriateness. Internal consistency reliability was assessed using Cronbach’s alpha coefficients. A pilot study involving 50 participants was conducted to test clarity and reliability; these participants were excluded from the final sample. The pilot findings indicated that no additional revisions were required.

Data collection started by 29 October till 24 December 2025 with an orientation session led by the researcher, during which the study aims and procedures were explained. Participants were informed that involvement was voluntary and were given the opportunity to ask questions. Ethical safeguards, including confidentiality measures, were clearly outlined to promote transparency and trust.

### 2.5. Statistical analysis

All collected data were reviewed for completeness and accuracy prior to entry into the statistical software. Data were analyzed using IBM SPSS Statistics. Descriptive statistics were used to summarize participants’ demographic characteristics and study variables. A p-value of less than 0.05 was considered statistically significant, while values of p ≤ 0.001 indicated highly significant results. To examine the proposed mediation relationships, path analysis was conducted using SPSS-AMOS version 26. Structural equation modeling (SEM) procedures were applied to test the hypothesized model. Confirmatory factor analysis (CFA) was conducted to assess model fit. The adequacy of the model was evaluated using multiple goodness-of-fit indices, including the chi-square (χ^2^) test and the χ^2^/df ratio, with values below 5 considered acceptable. Further fit criteria included the comparative fit index (CFI ≥ 0.95) and the root mean square error of approximation (RMSEA < 0.08) [28,29]. Data normality was tested using the Kolmogorov–Smirnov test, which confirmed a normal distribution; therefore, parametric statistical analyses were applied. Multicollinearity among the independent variables was examined using variance inflation factor (VIF) and tolerance values, where VIF values below 3 and tolerance above 0.10 indicated no multicollinearity issues. Continuous variables with normal distribution were reported as means and standard deviations (M ± SD). For group comparisons, independent samples t-tests were used for two groups, while one-way analysis of variance (ANOVA) was applied for comparisons involving three or more groups. Pearson’s correlation coefficient was used to explore relationships among key variables, including students’ digital competence, artificial intelligence readiness, digital literacy, and their respective subcomponents [30].

Data collection took place across various campus settings, including classrooms and library areas, between 10:00 a.m. and 2:00 p.m., Sunday through Thursday. Both verbal and written informed consent were obtained prior to participation. The questionnaire required approximately 10–15 minutes to complete. Participants were assured that all information would remain confidential and were informed of their right to withdraw from the study at any time without any consequences. Data were collected using a structured self-administered paper questionnaire, and distribution was scheduled in accordance with academic timetables to improve accessibility and enhance response rates.

### 2.6. Ethical considerations

Ethical approval was obtained from two universities for health sciences in Saudi Arabia and Egypt (IRB No. 00000241625; Approval No. 294), consequently. Before participation, individuals were provided with detailed information about the study and signed written informed consent forms. Strict measures were implemented to maintain anonymity and confidentiality throughout the research process. Participants were also clearly informed that their involvement was entirely voluntary and that they could withdraw from the study at any time without penalty or negative consequences.

## 3. Results

Participants’ sociodemographic and academic characteristics were assessed as shown in Table 1. Participants aged over 20 scored highest in digital competence (109.0 ± 14.4) and AI readiness (84.7 ± 12.8), with significant differences observed across age groups (p = 0.014 and p = 0.029, respectively). Male students demonstrated slightly higher digital literacy (64.0 ± 7.2) compared to females (p = 0.006). Employment status was associated with higher scores in digital competence (108.3 ± 14.3, p = 0.021) and digital literacy (64.0 ± 7.5, p = 0.007). Prior exposure to digital tools showed a strong positive relationship with all domains, particularly digital competence (109.4 ± 14.5 vs. 102.5 ± 14.6, p < 0.001) Similarly, frequency of digital tool use influenced outcomes, with participants who reported “always” using digital tools achieving the highest scores in digital competence (110.2 ± 15.0, p < 0.001) and AI readiness (84.9 ± 13.6, p = 0.014). Interest in emerging technologies demonstrated the most pronounced effect, as participants who were very interested scored 113.5 ± 13.3 in digital competence and 88.8 ± 12.2 in AI readiness (both p < 0.001). Parental education, especially at the university level, was associated with higher digital literacy, whereas residence, family type, and internet access did not show statistically significant differences.

**Table 1:** Participants’ Sociodemographic and Academic Characteristics and Their Association with Digital Competence, Digital Literacy, and AI Readiness among Study Participants (n = 500).

Variable	Category	Frequency	Percent	Digital competence	Digital literacy	AI Readiness
				**Mean ± SD**	**Mean ± SD**	**Mean ± SD**
Age	17-18	31	6.2	105.6 ± 16.6	62.8 ± 10.2	80.1 ± 16.1
	19-20	273	54.6	105 ± 14.9	62.5 ± 7.1	81.7 ± 13.4
	>20	196	39.2	109 ± 14.4	63.9 ± 7.1	84.7 ± 12.8
	***F* test**			**4.3(0.014)**	2.2(0.11)	**3.6(0.029)**
Gender	Male	243	48.6	107.9 ± 15	64 ± 7.2	83.5 ± 13.9
	Female	257	51.4	105.4 ± 14.8	62.2 ± 7.4	82.1 ± 12.9
	***t-*test**			1.85(0.065)	**2.78(0.006)**	1.14(0.27)
Work	Yes	229	45.8	108.3 ± 14.3	64 ± 7.5	83.6 ± 13.5
	No	271	54.2	105.2 ± 15.4	62.2 ± 7.2	82.1 ± 13.4
	***t-*test**			**2.32(0.021)**	**2.69(0.007)**	1.33(0.19)
Residence	Urban	101	20.2	105.3 ± 14.4	62.7 ± 7	81.4 ± 12.4
	Rural	399	79.8	107 ± 15	63.1 ± 7.5	83.1 ± 13.6
	***t-*test**			1.02(0.31)	0.48(0.63)	1.18(0.24)
Family type	Nuclear	332	66.4	107 ± 14.9	63 ± 7.4	82.9 ± 13.8
	Extended	168	33.6	105.9 ± 14.9	63.1 ± 7.3	82.5 ± 12.7
	***t-*test**			0.78(0.43)	0.22(0.83)	0.37(0.71)
Income	Not Enough	77	15.4	103.9 ± 18	61.5 ± 8.4	78.8 ± 14.8
	Enough	347	69.4	106.9 ± 14.3	63 ± 7.1	83.3 ± 13.2
	Enough &save	76	15.2	108.4 ± 14.2	64.6 ± 7.2	84.4 ± 12.2
	***F* test**			1.9(0.15)	**3.6(0.027)**	**4.2(0.016)**
Mother’s educational level	Illiterate	49	9.8	102.8 ± 18.4	60.6 ± 10	81.1 ± 15.5
	Basic	106	21.2	106.7 ± 14.6	62.8 ± 6.9	83.8 ± 12.9
	Secondary	189	37.8	105.4 ± 14.6	62.5 ± 6.7	81.8 ± 12.5
	University	156	31.2	109.2 ± 14	64.6 ± 7.1	83.8 ± 14.1
	***F* test**			**3.1(0.027)**	**4.6(0.003)**	1.07(0.36)
Father’s Educational Level	Illiterate	37	7.4	102.9 ± 16.6	61.5 ± 7.2	82.3 ± 12.5
	Basic	115	23.0	104.2 ± 16.3	61.8 ± 8.4	81.4 ± 14.3
	Secondary	161	32.2	106.6 ± 13.9	62.7 ± 6.9	82.9 ± 11.8
	University	187	37.4	108.9 ± 14.2	64.4 ± 6.9	83.7 ± 14.3
	***F* test**			**3.3(0.02)**	**4.08(0.007)**	0.72(0.54)
GPA	<2	25	5.0	99.6 ± 19.1	60.4 ± 11.8	76.6 ± 19.3
	2-2.9	127	25.4	105.6 ± 14.4	62.2 ± 6.6	83.1 ± 13
	3-3.9	322	64.4	107.2 ± 14.6	63.4 ± 7.1	82.8 ± 13.1
	4+	26	5.2	111 ± 15.3	64.7 ± 7.5	87.2 ± 10.6
	***F* test**			**2.9(0.031)**	2.39(0.068)	**2.8(0.039)**
Prior Exposure	Yes	302	60.4	109.4 ± 14.5	64.1 ± 7.3	84.7 ± 13.2
	No	198	39.6	102.5 ± 14.6	61.4 ± 7.2	79.8 ± 13.3
	***t-*test**			**5.2(0.000)**	**4.1(0.000)**	**4.02(0.000)**
Frequency of Use	Rarely	7	1.4	87 ± 24.4	52.9 ± 17.8	70.7 ± 25.1
	Occasionally	72	14.4	103.8 ± 15	62 ± 7.3	82.5 ± 14.7
	Frequently	271	54.2	105.9 ± 14	62.5 ± 6.4	82 ± 12.3
	Always	150	30.0	110.2 ± 15	65 ± 7.7	84.9 ± 13.6
	***F* test**			**8.2(0.000)**	**9.5(0.000)**	**3.55(0.014)**
Internet Access	Yes	468	93.6	107 ± 14.4	63.2 ± 7	83 ± 13.1
	No	32	6.4	101.8 ± 21.1	60.7 ± 11.6	79.8 ± 17.4
	***t-*test**			1.9(0.059)	1.2(0.23)	1.28(0.20)
Interest in Emerging Technologies	Not interested	46	9.2	100.9 ± 19.1	61.2 ± 10.4	78.4 ± 15.8
	To Somewhat	322	64.4	104.6 ± 14	62.4 ± 6.5	80.9 ± 12.8
	Very interested	132	26.4	113.5 ± 13.3	65.2 ± 7.7	88.8 ± 12.2
	***F* test**			**22.1(0.000)**	**8.6(0.000)**	**20.2(0.000)**
	Total	500	100.0			

Table 2 presents the bivariate Pearson correlation coefficients among digital competence, digital literacy, AI readiness, and their respective sub-dimensions for 500 participants. Digital competence shows strong, statistically significant positive correlations with all sub-dimensions, particularly with “Evaluate” (r = 0.845), “SMD” (Store, Manage, Delete; r = 0.842), and “CCS” (Communicate, Collaborate, Share; r = 0.786). Digital literacy is also strongly correlated with digital competence (r = 0.724) and its sub-dimensions, especially “Protect” (r = 0.678). AI readiness (MAIRS-MS) is highly correlated with digital competence (r = 0.749) and digital literacy (r = 0.663), and even more so with its own sub-dimensions: “Cognition” (r = 0.918), “Ability” (r = 0.932), and “Vision” (r = 0.833). All correlations are significant at the 0.01 level, indicating robust interrelationships among digital and AI-related competencies and their components.

**Table 2:** Bivariate correlations between study variables and sub-dimensions (n = 500).

Variables	Mean±SD	1	2	3	4	5	6	7	8	9	10	11	12	13
**1. Digital Competence**	106.62 ± 14.92													
2. SFA	19.65 ± 2.77	**.777** ^**^												
3. DAM	21.16 ± 4.48	**.782** ^**^	.493^**^											
4. CCS	11.65 ± 2.07	**.786** ^**^	.561^**^	.571^**^										
5. SMD	19.57 ± 3.20	**.842** ^**^	.652^**^	.524^**^	.646^**^									
6. Evaluate	22.47 ± 4.04	**.845** ^**^	.532^**^	.564^**^	.590^**^	.628^**^								
7. Protect	12.10 ± 2.08	**.724** ^**^	.576^**^	.323^**^	.513^**^	.636^**^	.634^**^							
8. Digital Literacy	77.97 ± 11.44	**.724** ^**^	.591^**^	.446^**^	.553^**^	.644^**^	.630^**^	.678^**^						
9.MAIRS_MS	82.78 ± 13.41	**.749** ^**^	.562^**^	.602^**^	.600^**^	.573^**^	.654^**^	.570^**^	**.663** ^**^					
10. Cognition	29.35 ± 5.64	**.669** ^**^	.442^**^	.625^**^	.531^**^	.473^**^	.590^**^	.451^**^	**.556** ^**^	**.918** ^**^				
11. Ability	30.69 ± 5.14	**.706** ^**^	.573^**^	.516^**^	.579^**^	.571^**^	.593^**^	.578^**^	**.656** ^**^	**.932** ^**^	.761^**^			
12. Vision	10.91 ± 2.31	**.635** ^**^	.455^**^	.541^**^	.483^**^	.442^**^	.600^**^	.451^**^	**.540** ^**^	**.833** ^**^	.707^**^	.728^**^		
13. Ethics	11.82 ± 2.05	**.568** ^**^	.505^**^	.310^**^	.465^**^	.512^**^	.488^**^	.527^**^	**.546** ^**^	**.732** ^**^	.543^**^	.666^**^	.549^**^	

**. Correlation is significant at the 0.01 level (2-tailed).

SFA = Search, Find, Access, DAM = Develop, Apply, Modify, CCS = Communicate, Collaborate, Share, SMD = Store, Manage, Delete, MAIRS-MS = Medical Artificial Intelligence Readiness Scale for Medical Students.

Table 3 and Fig 2 illustrate the mediating effect of digital literacy on the relationship between digital competence and AI readiness. They highlight both direct and indirect effects. The mediation analysis demonstrated that digital competence had a strong and significant positive effect on digital literacy (β = .813, B = .623, SE = .021, t = 29.737, p < .001), explaining 68.8% of the variance. In the direct effects model, both digital competence (β = .360, B = .323, SE = .045, t = 7.259, p < .001) and digital literacy (β = .457, B = .535, SE = .057, t = 9.352, p < .001) were significant predictors of AI readiness, with the model accounting for 63.5% of the variance. The total effect of digital competence on AI readiness was also statistically significant (β = .731, B = .657, SE = .029, t = 22.759, p < .001), explaining 57.0% of the variance.

**Table 3:** Direct, indirect, and total effects of digital competence on AI readiness mediated by digital literacy.

Models	B	β	*SE*	t	95% CI	P-value
**MV** **Model**						
Digital Competence → Digital Literacy	.623	.813	.021	29.737	.582 to.665	<.001
**Model Summary R2 = .688, F = 155.246, p < .001**						
**DV** **Model**						
Digital Competence → AI readiness	.323	.360	.045	7.259	.236 to.411	<.001
Digital Literacy→ AI readiness	.535	.457	.057	9.352	.423 to.648	<.001
Age (Covariate)	.863	.038	.631	1.366	−.378 to 2.103	.172
Income (Covariate)	.897	.037	.669	1.340	.181 to −.418	2.212
GPA (Covariate)	−.444	−.021	.593	−.749	−1.609 to.721	.454
Prior Exposure (Covariate)	−.087	−.003	.789	−.110	−1.636 to 1.463	.913
Frequency of Use (Covariate)	−.858	−.045	.539	−1.593	−1.917 to.201	.112
Interest in Emerging Technologies (Covariate)	1.238	.053	.666	1.858	−.071 to 2.547	.064
**Model Summary R2 = .635, F = 106.852, p < .001**						
**Total Effect**						
Digital Competence → AI readiness	.657	.731	.029	22.759	.600 to.714	<.001
**Model Summary R2 = .570, F = 93.236, p < .001**						
**Indirect effect**	**β**		**BootSE**		**BootLLCI**	**BootULCI**
Digital Competence → Digital Literacy→ AI readiness	.371		.052		.268	.475

B = unstandardized coefficient; β = standardized coefficient; SE = standard error; t = t-value; CI = confidence interval; LLCI = lower limit of confidence interval; ULCI = upper limit of confidence interval; p = probability value; R^2^ = coefficient of determination; F = F-statistic; MV = mediator variable model; DV = dependent variable model; BootSE = bootstrap standard error.

**Fig 2:**
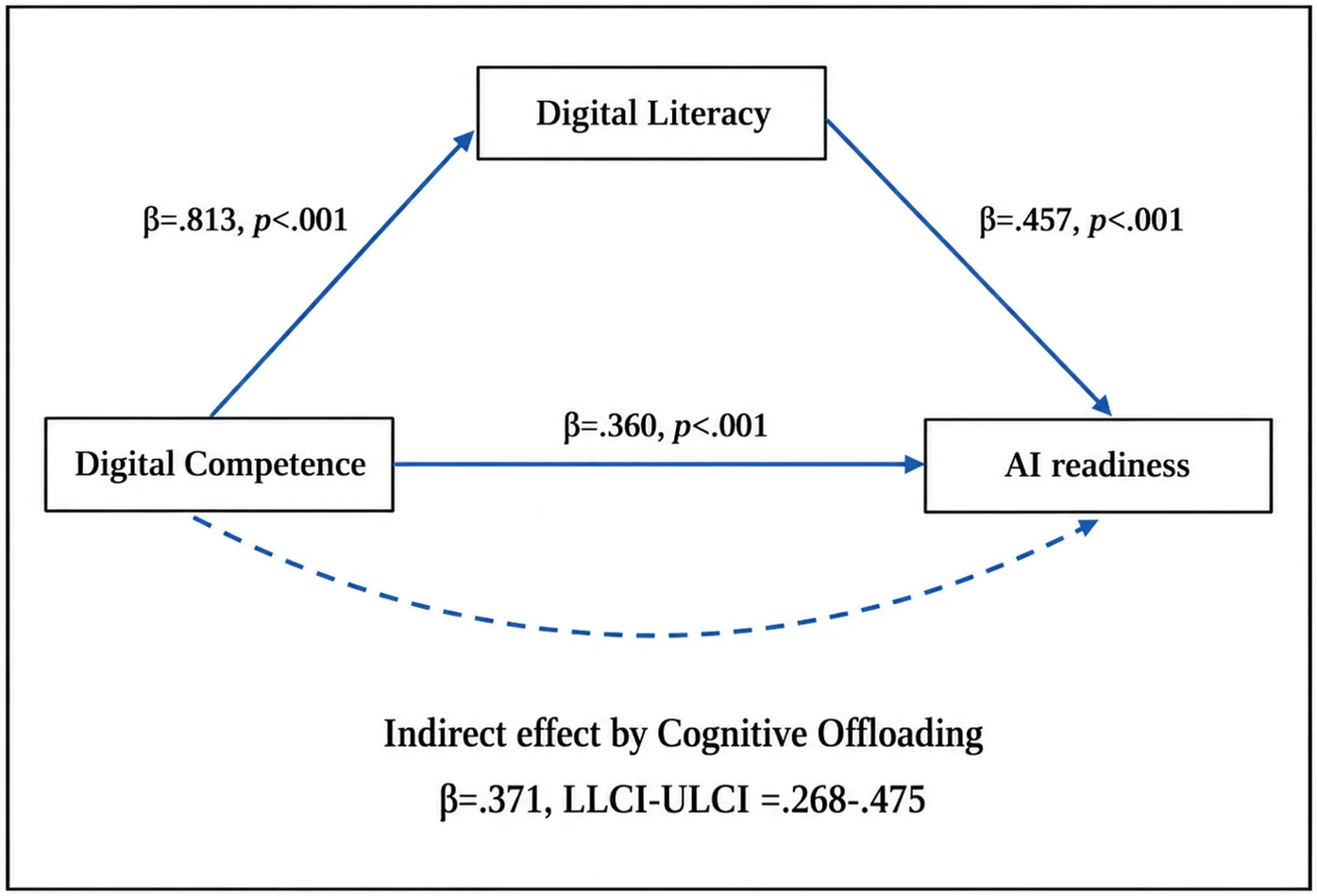
Mediating effect of digital literacy on medical AI readiness.

The structural model shows the direct and indirect relationships among digital competence, digital literacy, and medical AI readiness.

Bootstrapping analysis confirmed a significant indirect effect of digital competence on AI readiness through digital literacy (β = .371, BootSE = .052, 95% CI (.268,.475)), indicating partial mediation. Among the covariates, none showed statistically significant associations with AI readiness (p > .05), although interest in emerging technologies approached significance (p = .064). These findings suggest that digital competence influences AI readiness both directly and indirectly via digital literacy, highlighting the mediating role of digital literacy in this relationship.

## 4. Discussion

The strong positive correlation between AI readiness and digital competence among nursing students, as reported by Abou Hashish and Alnajjar (2024) [9] can be attributed to several interconnected factors. Digital competence forms the foundation for understanding and utilizing AI technologies, as it encompasses essential skills such as information literacy, technical proficiency, and data interpretation [11,12]. Students with higher digital skills are more capable of engaging with digital transformation services, which enhances their confidence and willingness to adopt AI tools in healthcare. Moreover, the study highlighted that students with strong digital competence also demonstrated commendable digital health literacy and positive attitudes toward AI, reinforcing the idea that digital proficiency supports both knowledge acquisition and attitudinal readiness. These relationships may be explained through technology acceptance and self-efficacy mechanisms, where higher digital competence enhances perceived ease of use and confidence in interacting with emerging technologies such as AI [17]. Consequently, students with stronger foundational digital skills are more likely to perceive AI systems as manageable and useful, which strengthens their readiness for adoption in clinical contexts. This suggests that digital competence functions not only as a technical skill set but also as a cognitive and psychological enabler of AI readiness [31].

These findings are echoed in the work of Asal et al. (2025) [32], who found significant positive correlations between digital competence, AI readiness, and pedagogical innovation among nurse educators, with digital competence being a strong predictor of AI integration in teaching practices. Similarly, Amin et al. (2025) [33] reported that digital competence positively influenced academic motivation and lifelong learning, both of which are essential components of sustained AI readiness. Together, these studies suggest that digital competence not only enables technical engagement with AI but also fosters the cognitive and emotional readiness needed to embrace AI-driven innovations in nursing education and practice [4].

The strong correlation between digital literacy and digital competence among nursing students, as observed in our findings, can be explained by the foundational and overlapping nature of these two constructs. Digital literacy is an essential component of broader digital competence. Our results are supported by existing literature that emphasizes the significance of digital literacy in shaping the readiness and capability of nursing students to engage with digital tools in both academic and clinical contexts. For instance, Lokmic-Tomkins et al. (2022) [34] identified gaps in first-year nursing students’ self-reported digital literacy despite frequent technology use, highlighting the need for targeted digital literacy education before clinical placement. They were more adept at performing essential tasks such as electronic documentation, digital communication, and information retrieval to support evidence-based practice. Similarly, Martzoukou et al. (2024) [24] highlighted deficits in specific areas of digital literacy such as information literacy, digital research, and innovation underscoring their importance for academic performance and professional preparedness. These components are integral to the development of overall digital competence. From a theoretical perspective, the overlap between digital literacy and digital competence reflects a hierarchical relationship in which digital literacy operationalizes core components of broader competence. This indicates that digital literacy may act as a functional pathway through which abstract digital skills are translated into practical capabilities required for academic and clinical performance in technology-enhanced healthcare environments.

Furthermore, Terry et al. (2019) [35] found that targeted interventions to improve digital literacy among nursing and midwifery students led to enhanced digital competence, especially when students engaged in peer-led initiatives like the NICE student champions program. These findings collectively suggest that digital literacy not only contributes directly to digital competence but also plays a pivotal role in enabling nursing students to confidently adopt digital technologies in their education and future practice. Therefore, fostering digital literacy is essential to building comprehensive digital competence among nursing students.

The strong positive correlation between AI readiness and digital competence/digital literacy among nursing students, as observed in our study, can be attributed to several interconnected factors, including the influence of key sociodemographic variables [16,36]. Our data revealed that prior exposure to digital tools was significantly associated with higher scores across all measured domains, most notably, digital competence. This suggests that students who had earlier and more frequent interactions with digital technologies entered nursing programs with a foundational familiarity that supported the development of both digital literacy and AI readiness. Frequency of digital tool use further amplified this relationship; students who reported consistently using digital tools (e.g., daily or always) demonstrated the highest levels of both digital competence and AI readiness, indicating that habitual engagement reinforces digital proficiency and confidence in using advanced technologies like AI.

Moreover, interest in emerging technologies emerged as the most influential factor, showing the strongest effect across all digital variables. Students who expressed a high level of enthusiasm for innovations such as artificial intelligence, digital transformation, and health informatics tended to outperform their peers in both digital literacy and AI readiness. This aligns with findings from Lokmic-Tomkins et al. (2022) [34], which emphasized that adequate digital literacy at the start of a nursing program is foundational for academic success and future professional practice. Similarly, Martzoukou et al. (2024) [24] noted that low competencies in key digital literacy dimensions, such as information literacy and digital research, could hinder students’ professional preparedness, further supporting the idea that strong digital literacy is integral to overall digital competence and AI readiness.

In addition, Terry et al. (2019) [35] and Baraka et al. (2025) [37] found that targeted initiatives to improve digital literacy, such as peer-led learning and structured support, positively influenced students’ digital competence. These findings reflect the dynamic and reinforcing relationship between sociodemographic influences (like prior exposure, frequency of use, and interest), digital literacy, and digital competence, all of which contribute significantly to AI readiness. Therefore, enhancing digital education early in the nursing curriculum and encouraging frequent, meaningful engagement with digital tools may be critical strategies to prepare students for the increasingly technology-driven landscape of healthcare [9,31,32]. These findings further suggest that digital readiness and AI preparedness are not solely skill-based constructs but are shaped by behavioral exposure patterns and motivational factors. Repeated engagement with digital tools may strengthen familiarity, reduce cognitive load when using technology, and increase adaptive learning behaviors, thereby facilitating smoother integration of AI-related competencies. These findings also highlight the importance of experiential learning opportunities, where repeated hands-on interaction with digital and AI-related tools enhances familiarity, reduces anxiety toward new technologies, and strengthens students’ confidence in applying AI in clinical and academic settings.

The present study revealed that digital literacy plays a mediating role in the relationship between digital competency and AI readiness, suggesting that individuals’ proficiency in basic digital skills serves as a bridge between their foundational technological capabilities and their preparedness to engage with artificial intelligence. This finding is consistent with prior research by Seker et al. (2025) [36], who identified digital literacy and data literacy as the strongest predictors of AI literacy. Their study further demonstrated that these literacies act as mediators between certain 21st-century skills and AI literacy, highlighting the layered nature of digital competencies in fostering AI readiness. Similarly, Muawanah et al. (2024) [21] emphasized the interconnectedness of digital literacy, artificial intelligence, and educational technologies, showing that AI-enhanced innovation, particularly in the context of language sustainability, was significantly shaped by AI readiness components such as cognition, vision, and ethics. Their results also indicated that digital literacy is crucial for interpreting and responding to the perceived threats and potentials of AI, especially in pedagogical contexts. Together, these findings support the assertion that while digital competency forms the foundation, digital literacy acts as a crucial transformative factor, enabling individuals to apply their skills effectively in AI-driven environments, ultimately enhancing their AI readiness. This mechanism can be further interpreted within the framework of layered competency development, where foundational digital competence supports the acquisition of digital literacy, which in turn enables higher-order cognitive processing required for AI readiness. This sequential pathway highlights that AI readiness is not a direct outcome of technical skills alone but is shaped through progressive cognitive and interpretive development.

## 5. Conclusion

This study highlights that digital literacy plays a significant mediating role in translating digital competence into medical AI readiness among undergraduate nursing students. Nursing students with strong digital competence and literacy are better prepared to engage ethically and effectively with AI technologies, supporting safe, informed, and technologically adept clinical practice. These findings emphasize the need to integrate comprehensive digital literacy training alongside technical digital skills within nursing curricula to develop a future-ready workforce capable of meeting the demands of AI-driven healthcare.

## 6. Limitations of the study

This study’s cross-sectional design limits causal inference between digital competence, digital literacy, and AI readiness. In addition, the use of self-reported data may introduce response bias. The sample was limited to undergraduate nursing students from two universities in Saudi Arabia and Egypt, and the sociodemographic characteristics may not represent the broader region, thereby restricting the generalizability of the results. The exclusion of students with prior AI training to minimize the exposure bias may have limited the inclusion of individuals with higher levels of AI readiness, which could potentially influence the variability of the results.

## Supporting information

S1 File Supporting_data_file_English. (XLSX)
